# Protection against Chemotaxis in the Anti-Inflammatory Effect of Bioactives from Tomato Ketchup

**DOI:** 10.1371/journal.pone.0114387

**Published:** 2014-12-31

**Authors:** Merel Hazewindus, Guido R. M. M. Haenen, Antje R. Weseler, Aalt Bast

**Affiliations:** Department of Toxicology, Faculty of Health, Medicine and Life Sciences, Maastricht University, Maastricht, The Netherlands; University Medical Center Freiburg, Germany

## Abstract

The consumption of tomato products has been associated with a decreased risk for chronic inflammatory diseases. In this study, the anti-inflammatory potential of tomato ketchup was evaluated by studying the effect of tomato ketchup extracts and bioactives from tomato ketchup on human monocytes and vascular endothelial cells (HUVEC). HUVEC were pre-treated for 1 h with either individual bioactives (7.5 µM lycopene, 1.4 µM α-tocopherol or 55 µM ascorbic acid) or a combination of these three compounds, or with the hydrophilic or lipophilic tomato ketchup extracts or with the two extracts combined. After the pretreatment, the cells were washed and challenged with TNF-α (10 ng/ml) for 6 h. The medium was used for the determination of the release of cytokines and the chemotaxis of monocytes. Inflammatory protein expression and production were assayed with real-time RT-PCR and ELISA. It was found that tomato ketchup extracts significantly reduced gene expression and release of the pro-inflammatory cytokines TNF-α and IL-8 in HUVEC after the inflammatory challenge, whereas the release of the anti-inflammatory cytokine IL-10 was increased. Chemotaxis was effectively impeded as demonstrated by a reduced monocyte migration. This effect correlated with the reduction of IL-8 production in the presence of the test compounds and extracts. The results consistently emphasize the contribution of lycopene to the anti-inflammatory effect of tomato ketchup. Other compounds in tomato ketchup such as α-tocopherol and ascorbic acid appeared to strengthen the anti-inflammatory effect of lycopene. The tomato ketchup extracts subtly interfered with several inflammatory phases that inhibit chemotaxis. Such a pleotropic mode of action exemplifies its potential mitigation of diseases characterized by prolonged low grade inflammation.

## Introduction

The consumption of tomatoes and tomato products is inversely associated with the risk for the development of inflammatory related diseases, such as atherosclerosis [1], [2]. The underlying pathology of these diseases is characterized by prolonged and low grade inflammation that ultimately leads to tissue damage and dysfunction [3].

Inflammation is triggered by e.g. intrusion of bacteria or tissue damage that results in the activation of nuclear transcription factors such as nuclear factor kappa B (NF-κB). NF-κB activation promotes the expression of several pro-inflammatory genes and the subsequent production and release of cytokines [4].

Cytokines are signaling molecules that mostly have a pro-inflammatory action (e.g. TNF-α, IL-6, IFN-γ). An important pro-inflammatory cytokine is TNF-α, which triggers a positive feedback loop during inflammation by activating NF-κB. Some cytokines display anti-inflammatory activity (e.g. IL-4, IL-10, IL-13) [5], [6]. Chemokines (e.g. monocyte chemotactic protein-1 (MCP-1), IL-8) are important for the recruitment of monocytes from the circulation to the site of inflammation. In this process, referred to as chemotaxis, the concentration gradient of the chemokine IL-8 is known to be of importance [7], [8].

An essential feature of chemotaxis is the up-regulation of adhesion molecules such as E-selectin and intercellular adhesion molecule-1 (ICAM-1) that are expressed on endothelial cells [9]. Receptors on the monocytes firmly adhere to these adhesion molecules, causing the monocytes to transmigrate through the endothelium. This migration is accompanied by differentiation of the monocytes into macrophages that phagocytose cell debris and bacteria. Chemotaxis leads to the accumulation of monocytes and macrophages in the vascular endothelium, which is the first hallmark of atherosclerotic plaque formation [8], [9]. Atherosclerosis contributes to life-threatening complications such as acute coronary disorders and myocardial infarction.

It has been reported that the consumption of tomatoes and tomato products like tomato juice, soup and paste, attenuate the inflammatory process and might protect against cardiovascular diseases [10], [11], [12], [13]. Tomatoes and tomato products contain various bioactive compounds such as carotenoids, flavonoids and vitamins. The carotenoid lycopene is the main constituent of tomatoes, followed by the vitamins α-tocopherol and ascorbic acid [14]. *In vitro*, α-tocopherol and ascorbic acid reduce oxidative stress [15], [16], [17]. *In vivo*, the antioxidant activity of α-tocopherol is less evident [18] and also anti-inflammatory effects have been proposed [19]. Therefore, these compounds are referred to as bioactives [20]. It should be noted that pathophysiological processes are often intertwined and that oxidative stress itself can initiate the inflammatory process by activating NF-κB and pro-inflammatory cytokines [21].

It has been observed that lycopene inhibits the activation of NF-κB [22], [23], the release of pro-inflammatory cytokines [23], [24] and prevents the expression of adhesion molecules *in vitro* [25]. Clinical studies support the anti-inflammatory potential of lycopene supplementation [10], [13], [25]. Furthermore, it was observed that the consumption of tomatoes and tomato products seemed to be more efficient against inflammation than lycopene supplementation. It was speculated that the presence of other bioactive compounds in tomato products contributed to this anti-inflammatory potential [11], [27].

Tomato products are frequently consumed in the Western World and a popular tomato product is tomato ketchup [28], [29]. There are no reports on the effect of tomato ketchup on inflammation. In the present study we examined the effect of tomato ketchup on a functional marker, i.e. in a bio-assay for chemotaxis involving transmigration of monocytes. To substantiate the results on chemotaxis, the sequential phases in the inflammatory process preceding chemotaxis namely gene expression and cytokine production, were assessed. The potency of tomato ketchup was estimated by assessing the anti-inflammatory effects of the hydrophilic and lipophilic extracts of tomato ketchup, and lycopene, α-tocopherol and ascorbic acid as individual compounds.

## Materials and Methods

### 1 Materials

Tomato ketchup was a gift from H.J. Heinz Company, the composition of the tomato ketchup is given in Table 1. The lycopene and α-tocopherol contents were determined after hexane extraction and reversed phase HPLC analysis. L-ascorbic acid was oxidized to dehydroascorbate using ascorbate oxidase, and dehydroascorbate was quantified using HPLC after reaction with o-phenylene diamine. L-ascorbic acid (CAS# 50-81-7) and RRR α-tocopherol (CAS# 59-02-9) were purchased from Sigma Chemical Co. (Germany). All-trans lycopene (CAS# 502-65-8) was supplied by Extrasynthese (France). All other chemicals were of analytical grade (>99% purity).

**Table 1:** The composition of the tomato ketchup used.

Lycopene	16.3 mg/100 g
α-Tocopherol	1.87 mg/100 g
Ascorbic acid	94.4 mg/100 g

### 2 Preparation of the tomato ketchup extracts and compounds

Lipophilic extracts of tomato ketchup were obtained by extracting 10 g tomato ketchup with 25 ml of chloroform, and subsequent centrifugation for 15 min. The chloroform was evaporated under a stream of nitrogen at room temperature to obtain a residue with the lipophilic compounds that were dissolved in 25 µl tetrahydrofuran, 25 µl heat inactivated fetal calf serum (FCS) and 950 µl F12K culture medium. The hydrophilic extract of tomato ketchup was obtained by adding 25 ml of F12K culture medium to 10 g tomato ketchup and subsequent centrifugation for 15 min. Lycopene and α-tocopherol were dissolved in chloroform, evaporated under a stream of nitrogen at room temperature and dissolved in 25 µl tetrahydrofuran, 25 µl FCS and 950 µl F12K culture medium. Ascorbic acid was dissolved in 10 ml F12K culture medium. The final concentrations of lycopene, α-tocopherol and ascorbic acid as single compounds and in the tomato ketchup extracts were 7.5 µM, 1.4 µM and 55 µM, respectively.

### 3 Cell culture

Immortalized human umbilical vein cells (HUVEC) (CRL 1730; American Type Cell Culture, USA) were cultured in F12K medium (Gibco, UK) containing 10% FCS (Gibco, UK), 100 units/ml penicillin (Gibco, UK), 100 µg/ml streptomycine (Gibco, UK), 20 mg endothelial cell growth supplement (BD Biosciences, UK) and 5 ml heparin (Leo Pharma, Belgium). Human monocytes U937 (CRL-1593.2; American Type Cell Culture, USA) were cultured in RPMI-1640 medium containing 200 mg/L L-glutamine and 25 mM HEPES (Gibco, UK), supplemented with 100 units/ml penicillin (Gibco, UK), 100 µg/ml streptomycin (Gibco, UK), 10% FCS in a 5% CO_2_ incubator at 37°C. HUVEC were grown to 90% confluence in 6-wells plates, the cells were washed with HBSS (Gibco, UK) and pre-treated for 1 h with either individual compounds (7.5 µM lycopene, 1.4 µM α-tocopherol or 55 µM ascorbic acid) or a combination of these three bioactives, or with the hydrophilic or lipophilic tomato ketchup extracts or with the two extracts combined (1∶1). For some compounds (lycopene, α -tocopherol) an organic solvent was used to prepare a solution of the compound. The proper volume of the solution was added first, and the organic solvent was completely evaporated under a stream of nitrogen. Subsequently, the final incubation mixture for the pretreatment was prepared. After the pretreatment, the cells were washed and challenged with TNF-α (10 ng/ml) for 6 h. The medium was used for the determination of the release of cytokines and for the chemotaxis assay. In the control samples only the solvent was used.

### 4 Cytokine quantification

TNF-α, IL-8 and IL-10 were quantified using PeliKine Compact human enzyme-linked immunosorbent assay (ELISA) kits (CLB/Sanquin, The Netherlands) based on appropriate and validated sets of monoclonal antibodies. Assays were performed as described in the manufacturer's instructions. Cytokine levels were related to those of the control incubation without tomato ketchup extracts or bioactive compounds. The tetrahydrofuran and FCS (both 0.05%) did not show any influence on the TNF-α induced cytokine release (data not shown).

### 5 RNA isolation, real-time reverse transcription polymerase chain reaction (qRT-PCR) and data analysis

Total RNA was isolated using Qiazol lysis reagent (Qiagen, The Netherlands) followed by purification using miRNeasy-kit (Qiagen, The Netherlands) according to the instructions of the manufacturer. RNA concentration and purity were measured using the Nanodrop system (IsoGen Life Science, The Netherlands). RNA purity was checked by dividing the absorbance of the extract at 260 nm by the absorbance at 280 nm, that was above 2 for all samples, and by dividing the absorbance of the extract at 260 nm by the absorbance at 230 nm, that was above 1.9 for all samples.

RNA isolation was performed on individual samples, using the iScript cDNA synthesis kit (Bio-Rad, The Netherlands). cDNA was synthesized from 0.5 µg RNA per sample and diluted 15× in RNase-free water. RT-PCR reactions were performed with iQ SYBR Green Supermix (Bio-Rad, The Netherlands) using the MyIQ single-color real-time PCR detection system (Bio-Rad, The Netherlands). Primers for the inflammatory markers TNF-α, IL-8, IL-10, E-selectin, ICAM-1, and IκBα (inhibitor protein kappa B) and the housekeeping gene β-actin were designed using Primer Express software (Applied Biosystems). Sequences of the primer pairs used were the following: for TNF-α 5′-TCAATCGGCCCGACTATCTC- 3′(forward), 5′- CAGGGCAATGATCCCAAAGT-3′(reverse), for IL-8 5′- GGACAAGAGCCAGGAAGAAA- 3′ (forward), 5′- AAATTTGGGGTGGAAAGGTT- 3′ (reverse), for IL-10 5′- GCTGTCATCGATTTCTTCCC- 3′(forward), 5′- CTCATGGCTTTGTAGATGCCT- 3′ (reverse), for e-selectin 5′- CCCTAGCAAGGCATGATGTT- 3′(forward), 5′- GGCCTCATGGAAGTTTTTCA-3′(reverse), for ICAM-1 5′- CTGAGCAATGTGCAAGAAGATAGC-3′(forward), 5′- CCCGTTCTGGAGTCCAGTACA-3′(reverse), for IKb 5′- CTACACCTTGCCTGTGAGCA-3′(forward), 5′- TCCTGAGCATTGACATCAGC-3′(reverse) and for β-actin 5′- CCTGGCACCCAGCACAAT-3′ (forward), 5′- CCTGGCACCCAGCACAAT-3′ (reverse). Then, 2.5 µl of 0.3 µM forward and reverse primer was mixed with 2.5 µl water and 12.5 µl SYBR Green Supermix (Bio-Rad, Hercules, USA) and added to 5 µl sample, yielding a final volume of 25 µl per well of a 96-well PCR plate. qRT-PCR was performed using a MyiQ Single Color real-time PCR detection system (Bio-Rad, Hercules, USA), according to the following protocol: denaturation at 95°C for 3 min followed by 40 cycles at 95°C (15 s) and 60°C (45 s). After PCR, a melt curve (60–95°C) was produced for product identification and purity. The PCR efficiency of all seven primer sets, assessed by measuring cDNA dilution curves, was at least 90%. Data were analyzed using the MyiQ software system (Bio-Rad, USA) and were finally expressed as relative gene expression (fold change compared to control samples) according to the 2^−ΔΔ*C*t^ method [30].

### 6 Chemotaxis assay

The chemotaxis assay was adapted from Yang et al. [31] and Denton et al. [32] with minor modifications. Briefly, after treatment with lycopene, α-tocopherol, ascorbic acid or tomato ketchup extracts and 24 h exposure to TNF-α as described in 2.3, the medium of the HUVEC was collected and transferred (800 µl) into the lower chamber of a transwell plate (5 µM pore size, Costar, USA). U937 cells were added to the insert (10^6^ cells/ml) and allowed to migrate through the polycarbonate membrane at 37C°, 5% CO_2_. After 90 min the cells in the lower wells were collected by centrifugation at 3000×*g* for 5 min and directly counted under a microscope.

### 7 Statistics

All experiments were performed, at least, in triplicate. Results are given as mean ± S.E.M. Since the data were not normally distributed, statistically significant differences between the experimental conditions and the control (containing neither tomato ketchup extracts nor lycopene, ascorbic acid or α-tocopherol) were assessd by a Kruskal-Wallis H test followed by Mann–Whitney *U* tests to locate post hoc potential differences between the groups. In order to correct for multiple pairwise post hoc comparison, the level of significance was set at *P*≤0.007 (Bonferroni corrected *P*-value for seven comparisons). In all other cases *P*≤0.05 was considered significant.

Relations between cytokine gene expression and protein levels under the various test conditions were appraised by calculating Pearson's correlation coefficient. The statistical analyses were performed by using SPSS15 for Windows (SPSS Inc. Chicago, USA).

## Results

### 1 Gene expression of inflammatory mediators in HUVEC

**Figure 1:**
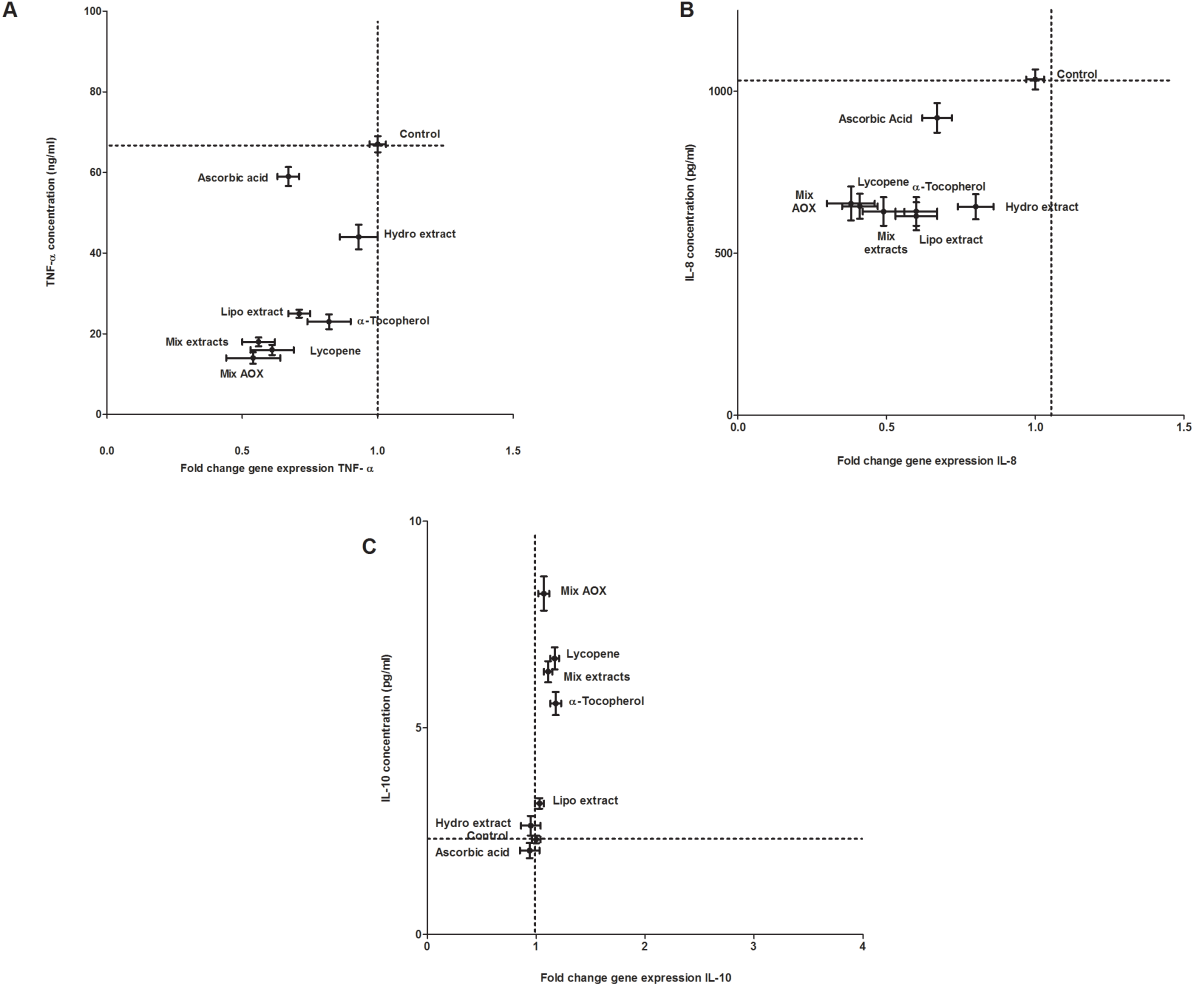
The effect of tomato ketchup extracts and lycopene (7.5 µM), α-tocopherol (1.4 µM) and ascorbic acid (55 µM) on gene expression of TNF-α (panel A) IL-8 (panel B) and IL-10 (panel C) and the concentration of these cytokines in the medium of the HUVEC after an inflammatory challenge. For TNF-α, the effect on gene expression showed a significant correlation with the effect on protein production (panel A). A comparable correlation was also seen for IL-8 (panel B), but not for IL-10 (panel C). Data are presented as mean ± S.E.M. of 3 triplicates. The effect of the pretreatments was significantly different (*P*≤0.05) from the control (only the inflammatory challenge), except for the hydrophilic extract on the gene expression of TNF-α, the effect of the hydrophilic extract and ascorbic acid on the IL-10 concentration, and the effect of all the pretreatments on the gene expression of IL10. Mix AOX: the combination of lycopene, α-tocopherol and ascorbic acid; Hydro: hydrophilic tomato ketchup extract; Lipo: lipophilic tomato ketchup extract; Mix extracts: the combination of the hydrophilic and lipophilic tomato ketchup extracts.

The gene expression of the pro-inflammatory cytokines TNF-α and IL-8 was reduced by the hydrophilic, lipophilic as well as the mixture of both tomato ketchup extracts (*P*≤0.05) (Fig. 1 a-b). The gene expression of the anti-inflammatory cytokine IL-10 was stimulated neither by the individual tomato ketchup extracts, nor by their combination.

**Figure 2:**
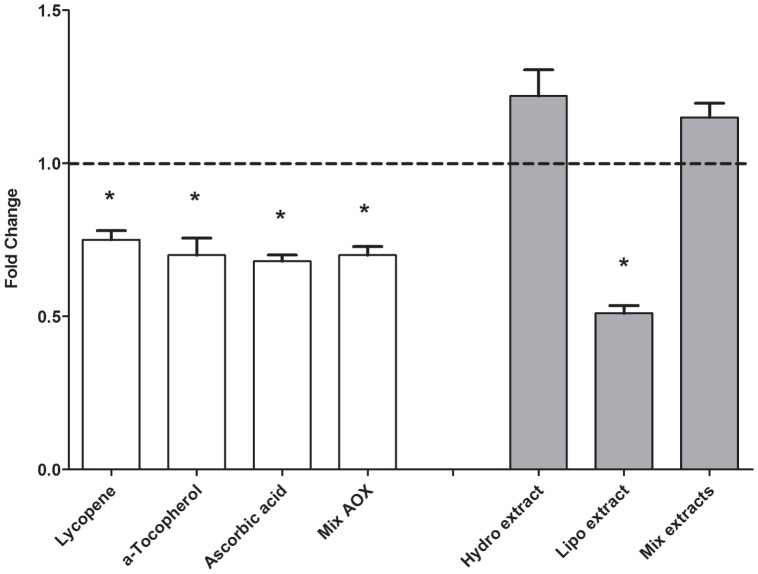
The effect of tomato ketchup extracts and lycopene (7.5 µM), α-tocopherol (1.4 µM) and ascorbic acid (55 µM) on gene expression of IκBα in HUVEC after an inflammatory challenge. Data are presented as mean ± S.E.M. of 3 triplicates,* significantly lower (*P*≤0.05) than the control (without extract or antioxidant). Mix AOX: the combination of lycopene, α-tocopherol and ascorbic acid; Hydro: hydrophilic tomato ketchup extract; Lipo: lipophilic tomato ketchup extract; Mix extracts: the combination of the hydrophilic and lipophilic tomato ketchup extracts.

**Figure 3:**
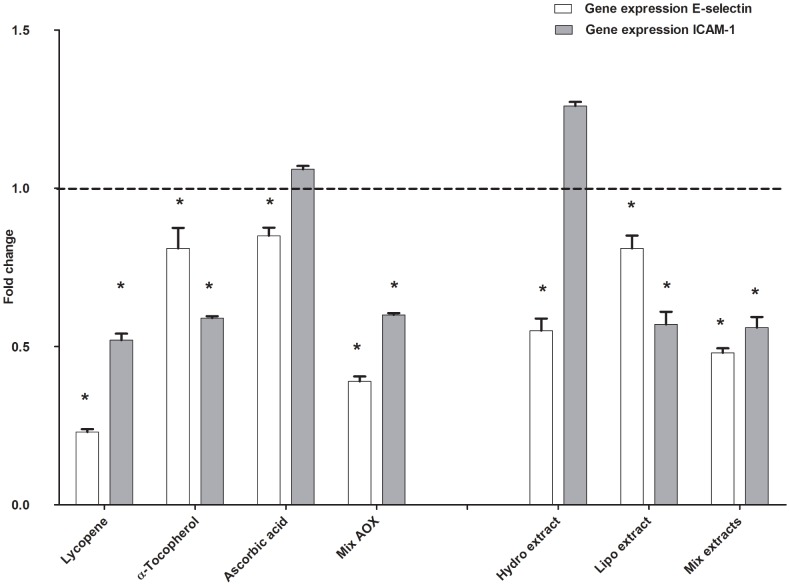
The effect of tomato ketchup extracts and lycopene (7.5 µM), α-tocopherol (1.4 µM) and ascorbic acid (55 µM) on gene expression of E-selectin and ICAM-1 in HUVEC after an inflammatory challenge. Data are presented as mean ± S.E.M. of 3 triplicates,* significantly lower (*P*≤0.05) than the control (only the inflammatory challenge). Mix AOX: the combination of lycopene, α-tocopherol and ascorbic acid; Hydro: hydrophilic tomato ketchup extract; Lipo: lipophilic tomato ketchup extract; Mix extracts: the combination of the hydrophilic and lipophilic tomato ketchup extracts.

The gene expression of the inhibitor protein IκBα was reduced by the lipophilic ketchup extract (Fig. 2). The gene expression of the adhesion molecule E-selectin was reduced by the hydrophilic, lipophilic ketchup extracts and the mixture of both extracts (Fig. 3). The lipophilic ketchup extract and the mixture of both extracts effectively decreased ICAM-1 gene expression (Fig. 3), while the hydrophilic extract exerted a slight stimulatory effect.

Lycopene, α-tocopherol, ascorbic acid and the mixture of these three bioactives reduced TNF-α and IL-8 gene expression (*P*≤0.05). Lycopene, α-tocopherol and the mixture of all three bioactives significantly stimulated the gene expression of the anti-inflammatory cytokine IL-10 (*P*≤0.05) (Fig. 1c). Lycopene, α-tocopherol, ascorbic acid and the mixture of all three bioactives effectively inhibited IκBα gene expression (Fig. 2). Lycopene, α-tocopherol and ascorbic acid, as well as the mixture of all three bioactives efficiently reduced E-selectin gene expression (Fig. 3). Lycopene and α-tocopherol and the mixture of all three bioactives significantly reduced the expression of ICAM-1, while ascorbic acid did not affect ICAM-1 gene expression (Fig. 3).

### 2 Cytokine production by HUVEC

Both tomato ketchup extracts inhibited the release of TNF-α and IL-8 in the culture medium (Fig. 1). The lipophilic tomato ketchup extract and the mixture of both extracts stimulated the release of the anti-inflammatory cytokine IL-10 in the culture medium (Fig. 1). For both TNF-α and IL-8, the ketchup extract induced inhibition of gene expression correlated with the attenuated secretion of the cytokines on protein level (Fig. 1 a-b).

Lycopene, α-tocopherol, ascorbic acid and the mixture of these three bioactives reduced the release of TNF-α and IL-8, and stimulated the release of IL-10 (*P*≤0.05). The release of TNF-α and IL-8 correlated with the decrease in gene expression, while the increased IL-10 secretion was not accompanied by a significant change in its gene expression (Fig. 1).

### 3 Chemotaxis bioassay

HUVEC were pre-treated with lycopene, α-tocopherol, ascorbic acid and extracts of tomato ketchup as described in section 2.6. After the inflammatory challenge, the culture medium was collected to determine the concentration of IL-8, and used to attract monocytes to the lower compartment of the transwell system in a chemotaxis bioassay.

**Figure 4:**
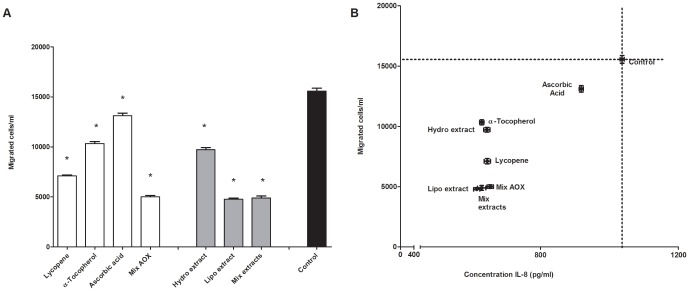
The effect of tomato ketchup extracts, lycopene, α-tocopherol and ascorbic acid on chemotaxis (panel A) and the relation between cell migration and IL-8 concentrations (panel B). Data are presented as mean ± S.E.M. of 3 triplicates. The effect of the pretreatments was significantly lower (*P*≤0.05) than the control (only the inflammatory challenge). The concentration of IL-8 showed a significant correlation with chemotaxis (R^2^ = 0.7316; *P*≤0.05). Mix AOX: the combination of lycopene, α-tocopherol and ascorbic acid; Hydro: hydrophilic tomato ketchup extract; Lipo: lipophilic tomato ketchup extract.

Both, the hydrophilic and in particular the lipophilic tomato extract significantly reduced the migration of monocytes. The combination of both extracts reduced migration by the same extent as the lipophilic extract (Fig. 4 a). α-Tocopherol, ascorbic acid and mainly lycopene significantly inhibited monocyte migration (Fig. 4 a). The results showed that a combination of lycopene, α-tocopherol and ascorbic acid inhibited chemotaxis even more than the individual bioactives.

A positive correlation (R^2^ = 0.7316, *P*≤0.05) between the migration of monocytes and the concentration of IL-8 in the culture medium was found (Fig. 4 b).

## Discussion

Prolonged, low-grade inflammation disturbs homeostasis and has been implicated in the etiology of chronic diseases such as atherosclerosis [1], [2], [5], [33]. Fast food and unbalanced diets increase the risk for cardiovascular diseases [34], [35]. Evidence is accumulating that fruits and vegetables provide substantial health benefits, especially against these chronic diseases [36], [37], [38], [39]. It has been reported that tomatoes and tomato products exert anti-inflammatory effects [1], [33], [40]. In the US the frequent tomato ketchup consumption among people aged between 15–35 years is responsible for their relatively high lycopene intake [41]. This prompted us to investigate the effect of tomato ketchup extracts on a functional marker of a pivotal process during inflammation, i.e. chemotaxis involving the transmigration of monocytes into the vascular wall. The sequential phases in the inflammatory process preceding chemotaxis namely gene expression and cytokine production, were also assessed. Interestingly, a consistent inhibitory effect of tomato ketchup extracts on the various phases of the inflammatory process was observed.

We assessed the activity of NF-κB via the expression of the IκBα gene. IκBα gene expression is directly related to NF-κB activation [4], [42], [43], [44]. The lower IκBα gene expression indicated that the lipophilic tomato ketchup extract, as well as the individual bioactives tested, attenuated the initial phase of the inflammation process by reducing the activation of the transcription factor NF-κB. The hydrophilic extract had a relatively low anti-inflammatory potency and even tended to display a pro-inflammatory activity. The sugar in the hydrophilic tomato ketchup extract might be involved in the observed slightly increased IκBα expression. It appeared that the lipophilic extract most effectively inhibited the expression of IκBα (Fig. 2).

The effect on IκBα gene expression was consistent with the observed inhibition on gene expression of the pro-inflammatory cytokines TNF-α and IL-8 by tomato ketchup extracts that is controlled by NF-κB. In line with the reduced gene expression, the release of TNF-α and IL-8 was inhibited with 61±9% and 69±4%, respectively, by the tomato ketchup extracts. Of the bioactive compounds tested, lycopene was found to be the most effective.

TNF-α incites the inflammatory process by a positive feedback loop since TNF-α itself can activate NF-κB [4], [45]. As a consequence of NF-κB activation, the transcription and release of TNF-α increase which further amplifies the inflammatory process. The inhibitory effect of tomato ketchup extracts on TNF-α release is expected to interrupt this positive feedback loop involving NF-κB.

During the course of inflammation also anti-inflammatory cytokines such as IL-4, IL-10 and IL-13 are formed. [5]. These cytokines initiate a negative feedback loop. The combined tomato ketchup extracts appeared to increase IL-10 production, indicating stimulation of the negative feedback mechanism. The effect on the protein production of IL-10 was relatively high while gene expression remained largely unaffected. This might suggest the anti-inflammatory feedback control by IL-10 is affected via another mechanism than its gene expression.

There was a good correlation between the gene expression and cytokine production of TNF-α and IL-8 (Fig. 1). This suggests that modulating gene-expression is involved in the reduction of the production of these pro-inflammatory cytokines by tomato ketchup extracts. Apparently, the anti-inflammatory effect of tomato ketchup extracts is achieved via different molecular modes of actions. These results also indicate that tomato ketchup mitigates inflammation in an early phase by two distinct mechanisms i.e. by inhibiting the pro-inflammatory positive feedback loop and by stimulating the anti-inflammatory feedback loop. In both processes, lycopene again seems to be the most effective compound.

A crucial event of the inflammatory process is the migration of monocytes from the vascular lumen into the vascular wall. This process, known as chemotaxis [5], [6], [8] consists of sequential interactions between monocytes and endothelial cells. Firstly, endothelial cells are triggered by chemotactic factors (e.g. IL-8) to express adhesion molecules such as E-selectin and ICAM-1 [7]. Consistent with the inhibition of IL-8 release, our results showed that tomato ketchup extracts reduced the expression of E-selectin and ICAM-1 (Fig. 3). After the monocytes adhere to the adhesion molecules on the endothelium, the monocytes migrate to the inflamed site, which is initiated by a concentration gradient of chemotactic factors such as IL-8 [7]. In this study the culture medium of inflammatory challenged endothelial cells, which released IL-8, was used in a bioassay for the determination of monocytes chemotaxis. The culture medium of tomato ketchup extracts treated endothelial cells reduced the chemotaxis of human monocytes effectively, by 69±4%. The reduced chemotaxis correlated significantly with the decreased concentration of IL-8 in the medium that originated from the exposed endothelial cells (Fig. 4). These observations demonstrate the potential of tomato ketchup extracts to avert chemotaxis.

Tomato ketchup is a mixture in which lycopene, α -tocopherol and ascorbate are the most studied ingredients. It contains also numerous other bioactives e.g. carotenoids other than lycopene and flavonoids. Our results consistently underline the contribution of lycopene to the anti-inflammatory effect of tomato ketchup. α-Tocopherol and ascorbic acid appeared to strengthen the anti-inflammatory effect of lycopene. These findings are in line with our previous *in vitro* results which also indicate that the inflammatory process was mitigated by especially lycopene, and that a combination of α-tocopherol and ascorbic acid synergistically prevented oxidative damage [24]. Together these compounds act on different levels within the inflammatory process which complements their anti-inflammatory potential [24]. In the present study, it was observed that the mixture of lycopene, α-tocopherol and ascorbic acid inhibited chemotaxis of monocytes. However, the inhibitory effect of tomato ketchup extracts appeared to be even more potent, demonstrating that compounds not identified in the present study also contribute to the anti-inflammatory effect of tomato ketchup extracts.

Evidence on the anti-inflammatory properties of tomato ketchup is scarce. To our knowledge this is the first study that addresses this subject on the various phases of the inflammatory process in a structured and detailed approach. The effects of lycopene are much better documented than the effects of tomato products. For example, the anti-inflammatory potential of lycopene has been described in the study of Hung et al. who reported that lycopene treatment of endothelial cells significantly inhibited NF-κB activation and expression of ICAM-1 in endothelial cells [25]. Di Tomo et al. demonstrated a significant reduction of TNF-α formation in lycopene treated human endothelial cells [46] . Both, Feng et al. [47] and Palozza et al. [22] showed that lycopene attenuated IκBα phosphorylation in macrophages. Additionally, lycopene also effectively reduced the production of pro-inflammatory cytokines *in vitro* [22], [47]. In line with previous observations, our study also emphasizes that lycopene plays an essential role in the anti-inflammatory effects of tomato ketchup [1], [2], [12], [14], [51].

Moreover, the results of the present study draw attention to the importance of compounds in tomato ketchup other than lycopene. For instance, Djuric et al. [48] demonstrated that processed foods had a higher antioxidant capacity compared to fresh foods. Surprisingly, processed foods with the highest protective potential did not contain the highest lycopene levels [48]. In this respect, also the anti-inflammatory effects of α-tocopherol and ascorbic acid have to be taken into account. In isolation, these bioactives reduce the production of pro-inflammatory cytokines like TNF-α [49], [50], [24]. Also other bioactives have been identified in the tomato ketchup (e.g. phytoene and phytofluene, precursors to lycopene [52]). In clinical settings, the anti-inflammatory effects of tomatoes and tomato products have also been studied [10], [13], [51], [53]. In the study of Sanchez-Moreno et al. the effect of gazpacho, a vegetable soup containing high levels of tomatoes, on pro-inflammatory mediators in healthy volunteers was examined. It was found that the combination of various bioactives in this soup protected against oxidative stress and inflammation by reducing the generation of prostaglandins and chemoattractants [10]. Blum et al. did not observe an anti-inflammatory effect after the consumption of a tomato-rich diet in healthy volunteers [26]. However, in the study of Riso et al. the production and release of TNF-α was effectively reduced after the consumption of tomato juice [13]. Similarly, Markovits et al. reported that supplementation of tomato-derived lycopene, attenuated pro-inflammatory markers including TNF-α, IL-6 and C-reactive protein in obese patients [40]. The majority of studies that investigated the effects of tomato products concluded that the participation of bioactive compounds other than lycopene, also contributed to the observed health effects [10], [11], [54].

The findings of the present study highlight the anti-inflammatory potential of tomato ketchup extracts. The effects on specific pathways seem to be relatively subtle and will not result in a complete block of the individual inflammatory phases. Tomato ketchup extracts appear to interfere on several early levels of the inflammatory process by inhibiting positive and stimulating negative regulatory feedback loops. These effects become integrated in a clear inhibition of chemotaxis. Such a pleotropic mode of action can especially be effective in mitigation of prolonged, low-grade inflammation. Based on the results of the current study, tomato ketchup seems to have the potential to contribute to the protection against health conditions associated with low-grade inflammation, which deserves further attention.
